# Identification of community-dwelling older adults at risk of frailty using the PERSSILAA screening pathway: a methodological guide and results of a large-scale deployment in the Netherlands

**DOI:** 10.1186/s12889-019-6876-0

**Published:** 2019-05-03

**Authors:** Stephanie Jansen-Kosterink, Lex van Velsen, Sanne Frazer, Marit Dekker-van Weering, Rónán O’Caoimh, Miriam Vollenbroek-Hutten

**Affiliations:** 1 Roessingh Research and Development, Roessinghsbleekweg 33b, 7522 AL Enschede, The Netherlands; 2 University of Twente, Faculty of Electrical Engineering, Mathematics and Computer Science, Telemedicine group, Enschede, the Netherlands; 3 ZiekenhuisGroep Twente (ZGT), scientific office ZGT academie, Almelo, the Netherlands; 4 Centre for Gerontology and Rehabilitation, University College Cork, Cork City, Ireland; 5 Clinical Sciences Institute, National University of Ireland Galway, Galway City, Ireland

**Keywords:** Older adults, Primary prevention, Frail, Pre-frail, Screening, Outcome

## Abstract

**Background:**

Among community-dwelling older adults, frailty is highly prevalent and recognized as a major public health concern. To prevent frailty it is important to identify those at risk of becoming frail, but at present, no accepted screening procedure is available.

**Methods:**

The screening process developed as part of the PERSSILAA project is a two-step screening pathway. First, older adults are asked to complete a self-screening questionnaire to assess their general health status and their level of decline on physical, cognitive and nutritional domains. Second, older adults who, according to step one, are at risk of becoming frail, are invited for a face-to-face assessment focusing on the domains in depth. We deployed the PERSSILAA screening procedure in primary care in the Netherlands.

**Results:**

In total, baseline data were available for 3777 community-dwelling older adults (mean age 69.9 (SD ± 3.8)) who completed first step screening. Based on predefined cut-off scores, 16.8% of the sample were classified as frail (*n* = 634), 20.6% as pre-frail (*n* = 777), and 62.3% as robust (*n* = 2353). Frail subjects were referred back to their GP without going through the second step. Of the pre-frail older adults, 69.7% had evidence of functional decline on the physical domain, 67% were overweight or obese and 31.0% had evidence of cognitive decline.

**Conclusion:**

Pre-frailty is common among community-dwelling older adults. The PERSSILAA screening approach is a multi-factor, two-step screening process, potentially useful for primary prevention to identify those at risk of frailty and who will benefit most from preventive strategies.

## Background

Demographic ageing is a global trend. In the European Union, the number of people aged 65+ will almost double over the next 50 years, from 85 million in 2008 to 151 million in 2060 [1]. Among these older adults, frailty is highly prevalent; it is also recognized as a major public health concern by the World Health Organization [1]. At the moment, there is, however, no consensus on a definition for frailty [2–4]. Nonetheless, Vellas & Sourdet, 2017 [5] claim that there is an emerging consensus that preventing frailty in older adults could improve health outcomes and quality of life, and enables a longer period of independent living. In short, it is important to prevent older adults becoming frail [5, 6]. As preventing frailty starts with identifying older adults at risk of becoming frail (pre-frailty), early screening among at risk populations is important [7, 8] and multiple tools are used by clinicians to assess frailty [9]. However, since there is no golden standard [10], the search for the optimal tool continues. An ideal tool to assess those at risk should be easily applicable in daily clinical practice and should be able to assess multiple elements of pre-frailty or frailty including physical, psychological and social domains [11]. It is therefore problematic that most screening instruments only assess the presence of frailty and cannot single out those older adults who are at risk of becoming frail [12, 13]. This relates to the current lack of a definition of pre-frailty, which is most often defined by cut-offs on frailty screening instruments not reaching the threshold for frailty. Instead, pre-frailty is a complex multi-dimensional risk-state before onset of frailty characterised by pathophysiological changes which can result in poor health outcomes (unpublished systematic review).

In order to intervene promptly, appropriate screening procedures to identify older adults at risk of becoming frail, combined with a set of tailored interventions, are required [14]. The aim of the PERSSILAA (Personalised ICT Supported Service for Independent Living and Active Ageing) project (FP7-ICT-610359) was to develop a community-based, technology-supported health service model that aims to screen older adults for pre-frailty to prevent the development of frailty [15]. As physical and cognitive decline as well as malnutrition, have been identified as major elements of frailty among older adults [16], this project focused on improving these three domains.

The aim of this article is to present the PERSSILAA screening model and the outcome of the screening process. In the Methods section, the two-step PERSSILAA screening pathway will be described in detail including the set-up of population-level (pre)frailty screening in the east of the Netherlands (Twente region). In the Results section, the cross-sectional outcomes of the PERSSILAA screening will be presented including prevalence data and an analysis of the merit of the individual screening instruments that were used to identify (pre) frailty. The Discussion section, finally, will discuss the implications of our work, both in terms of inferences for public health and for population-level screening for frailty.

## Methods

### The PERSSILAA screening procedure

The PERSSILAA screening pathway was a two-step annual screening programme to identify older adults at risk for developing frailty (i.e. pre-frailty). First, older adults were asked to complete a self-screening questionnaire to assess their general health status and their level of decline on physical, cognitive and nutritional frailty domains. Second, older adults who, according to step one, were at risk of developing frailty were invited for a face-to-face assessment that focuses on the physical, cognitive and nutritional domains in more depth.

During the first step, older adults with an age between 65 and 75 years old were asked by their General Practitioner (GP) to complete a comprehensive set of questionnaires including questions on health status and demographic characteristics. This was done via a mass mailing (postal survey) to ensure that all older adults within defined GP practices were reached. These GPs signed up to participate after a general meeting of GPs organized by the municipality. Prior to posting the PERSSILAA screening questionnaire to patients, GPs identified those patients who were, in their opinion, too frail to participate. These patients included those with multiple serious illnesses or those with limited life expectancy monitored extensively by their GP; these did not receive an invitation. Based on the preferences of the older adult, questionnaires can be completed on paper or online. Previous research has shown that offering different types of administration (online or on paper) does not affect screening results [17, 18]. When the questionnaire was completed online, older adults could receive their results immediately. When the questionnaire was completed on paper, adults could hand over the questionnaire at their GP office or send it by regular mail. Afterwards they received their results via email or by regular mail (when their email address was unknown). Older adults screening as robust were not contacted further; older adults screening positive for pre-frailty were invited for a face-to-face screening (Step two of the PERSSILAA screening); older adults classified as frail, were contacted by their GP [15, 19].

During step two of the PERSSILAA screening, a face-to-face assessment was conducted aiming to gain more information on the level of functional decline on physical, cognitive and nutritional domains. During the PERSSILAA project the face-to-face assessments were performed by trained volunteers or students at a location in the older adult’s neighbourhood (e.g., a local retirement home). After the face-to-face meeting, older adults received the outcomes of their assessments, information about healthy ageing and advice about the various health programs available in their community. Older adults that were identified as pre-frail on one of the three domains were offered existing training services targeting physical and cognitive domains available in their community organized by their municipality or local welfare organisation, and education about healthy eating for older adults. The service model that accompanies the PERSSILAA screening model was the result of a participatory design process, in which all stakeholders were actively involved. Among other things, it was decided to limit the age span of the screening program from 65 to 75 years of age, in order to not interfere with existing screening programs for those over 75 years old and to take advantage of the technology skills of the less old cohort. For more information about the development of the model we refer to Van Velsen et al., 2015 [15].

### Instruments including in step one of the PERSSILAA screening pathway

The initial instrument scored was the Dutch version of the Groningen Frailty Indicator (GFI) [20, 21] administered to assess the older adult’s general level of frailty. The GFI is a validated 15-item screening instrument that measures loss of function and resources across physical, cognitive, social and psychological domains. Each item is rated on a 3-point scale (Yes, No, Sometimes). The total score of the Groningen Frailty Indicator has a range from 0 to 15. A score of 4 or higher represents moderate to severe frailty. The SF-36 [22] physical functioning subscale (PF-10) was then administered to examine perceived physical functioning. The PF-10 consists of 10 items, each rated on a 3-point scale (yes, limited a lot; yes, limited a little; and no, not limited at all). In order to calculate an overall score, all answers are summed and then transformed to a 0–100 scale. Higher scores represent better health status. Older adults are classified as limited in physical functioning if they score below a cut-off score of 61 (pooled mean score in a general older Dutch population) [23]. The SF-36 PF-10 is validated in Dutch [24]. To screen for cognitive impairment, the self-administered version of the AD8 Dementia Screening Interview (AD8) [25, 26] was used to detect early cognitive changes associated with many common dementing illnesses. Older adults were asked to rate changes in their ability for each of the 8 items, without attributing causality. Each item is rated on a 3-point scale (Yes, a change; No, no change; and Don’t know). The final score is a sum of the number items marked “Yes, a change”. Based on clinical research findings and validation samples, the following cut points are provided: 0–1: Normal cognition and 2 or greater: cognitive impairment is likely to be present. The AD8 is not validated in Dutch. Finally, the Mini Nutrition Assessment Short-Form (MNA-SF) [27] was administered to identify those who were malnourished or at risk of malnutrition. The MNA-SF consist of 6 items, focusing on food intake, weight loss, mobility, psychological stress, neuropsychological problems and Body Mass Index (BMI). By summing up the scores on all 6 items, a total score is derived. A score of 12 to 14 points indicates a normal nutritional status, a score of 8 to 11 points indicates risk of malnutrition and a score of 0 to 7 points indicates that the participant is malnourished. The MNA-SF is validated [28], but not in Dutch.

Based on the GFI, older adults were initially stratified as either robust, pre-frail or frail. GFI scores higher than 4, were classified as frail; scores of 4 were classified as pre-frail [20]. Older adults were also screened and categorised based on deficits in physical, cognitive and/or nutritional domains identified from the other screening instruments. Older adult were also assigned to the pre-frail group irrespective of their GFI score when they scored 60 or below on the PF-10 items of the SF-36 and / or when they scored 2 or higher on the AD8 and / or if their score on the MNA short form was 7 or lower. Finally, older adults that did not meet at least one of the conditions for being classified as pre-frail or frail were classified as robust. Table 1 provides an overview of the screening questionnaires used during the first step of the PERSSILAA screening procedure and the triage to the three groups.

**Table 1:** Overview of the questionnaires of step one of the PERSSILAA screening and triage to the three groups

Questionnaire	Domain	Outcome
Groningen Frailty Indicator (GFI) [20, 21]	General level of frailty	4 = pre-frail ≥ 5 = frail
SF-36 [22] physical functioning scale (PF-10)	Physical domain	≤ 60 = functional decline/physical pre-frailty
The AD8 Dementia Screening Interview (AD8) [25, 26]	Cognitive domain	≥ 2 = cognitive impairment/cognitive pre-frailty
Mini Nutrition Assessment Short-Form (MNA-SF) [27]	Nutritional domain	≤ 7 = possible malnutrition/nutritional pre-frailty

### Instruments including in step two of the PERSSILAA screening pathway

To assess the physical status of older adults four tests were used. First, the timed up and go (TUG) test [29] was administered. The TUG test is a simple office-based test, used to identify persons at risk of falling because of balance or gait problems. The TUG test is performed 3 times and an average score is calculated. Second, the chair stand test (CST) [30] was administered. The CST tests the level of lower extremity strength. Third, the chair sit and reach test (CSRT) [31] was administered. The CSRT assess the level of physical flexibility. Fourth and finally, the two-minute step test (2MST) [32] was administered. The 2MST tests the level of aerobic endurance. All four objective tests have normal range scores related to gender and age based on previous research [32]. Scoring for TUG test was adapted to the range scores of Rikli & Jones [32], where an 8-ft TUG test was performed instead of 3 m. Scoring of the CSRT is indicated in inches to compare the score with the normal range. Scoring below the pre-defined cut-off scores indicates “functional decline”.

For the assessment of the cognitive status, the Quick Mild Cognitive Impairment (Q*mci*) screen was used. The Q*mci* screen is a short screening test for cognitive impairment, developed as a rapid, valid and reliable tool for the early detection and differential diagnosis of mild cognitive impairment (MCI) and dementia [33]. The Qmci screen has six subtests covering the following cognitive domains: orientation, working memory (registration), visuospatial/executive function (clock drawing), semantic memory (verbal fluency), and two episodic memory tests (delay recall and logical memory) [34]. Validated in Dutch [35], the Q*mci* screen can be completed in 3 to 5 min [34]. The overall Q*mci* screen score ranges from 0 to 100, with lower scores indicating cognitive impairment [36].

For the assessment of the nutritional status, the extended form of the Mini Nutrition Assessment (MNA) [27] and waist circumference were used. The extended version of the MNA is a validated screening tool that identifies older adults who are malnourished or at risk for malnutrition. In addition the items of the MNA-SF, the full MNA consists of an extra 12 items to provide additional information. The overall score of the full MNA is calculated by summing the scores on all 18 items.

### Ethics

Considering to Dutch law (Medical Research Involving Human Subjects Act), the nature of this research (general screening of older adults by questionnaire and face-to-face screening) and that public screening initiated by GPs for different health conditions is usual care in the east of the Netherlands, this study did not require formal ethical approval. The appropriate ethics committee (METC Twente) ruled that no formal ethics approval was required in this particular study (K14–42). At the end of the questionnaire, older adults were asked whether they agreed to the use of their data for research purposes, when they agreed they had to tick a checkbox. Only the data from those consenting were included in the PERSSILAA screening database. All data were anonymized.

### Statistical analyses

Statistical analyses were performed with IBM SPSS Statistics 19 for Windows. All outcome measures were visually inspected for normal distribution using histogram and probability plots, prior to the selection of appropriate statistical tests. Descriptive statistical methods were applied for each of the outcome measures. Normal data were presented as mean ± standard deviation (SD), non-normal as median with range. As the PERSSILAA screening was an annual screening during the length of the PERSSILAA project (three years), only the baseline (first) complete two-step screening data were analyzed and presented in this paper. For statistical analyses, the level of significance was set at α < 0.05.

## Results

### Demographics

The PERSSILAA project stated in November 2013 and ended November 2016 and this study was carried out in year two and three of this project. In total, 32 GP offices participated and 10,331 community-dwelling older adults were invited to participate in the first step of the PERSSILAA screening. With a response rate of 36.6%, the data of 3777 of the older adults were stored in the PERSSILAA screening database. The demographic characteristics of the 3777 older adults included in this analysis are presented in Table 2. The mean age of the older adults was 69.9 (SD ± 3.8). The majority of those participating had access to the internet, and 30% of the older adults completed the questionnaire online.

**Table 2:** Demographic characteristics of the baseline PERSSILAA screening sample (*n* = 3777)

Gender (Percentage)	48.3% male
	51.7% female
Age (Mean years and Standard deviation, SD)	69.9 (SD ± 3.8)
Body Mass Index (Mean and standard deviation, SD)	27.2 (SD ± 4.7)
Level of Education (Percentage)	1.8% No qualification
	9.9% Primary school
	17.9% Secondary school
	22.6% Vocational school for 2–3 years
	25.3% High school
	18.2% Bachelor’s degree
	4.3% University / PhD
Living situation (Percentage)	20.9% alone
	79.1% with someone else
Internet access (Percentage)	88.3% yes
	11.7% no
Alcohol – daily intake (Percentage)	51.3% yes
	48.7% no
Smoking (Percentage)	11.8% yes
	88.2% no
Number of cigarettes (Mean years and Standard deviation, SD)	9.6 (SD ± 6.7)

### Results of step one of the PERSSILAA screening

Based on the first step of the PERSSILAA screening, 16.8% of the older adults were classified as frail (*n* = 634), 20.6% of the older adults were classified as pre-frail (*n* = 777), and 62.3% of the older adults were classified as robust (*n* = 2353). The results of 13 participants were incomplete. Table 3 provides an overview of the average score on each measurement instrument according to the classification of the participants. Among the older adults identified as frail, 11.3% showed impairments in all three domains, 24.6% had decline on two of the three domains (16.9% in the physical and cognitive domains, 4.8% in the physical and nutritional domains and 2.9% on the cognitive and nutritional domains), and 36.8% experienced problems in only one domain (21.7% in the physical domain, 11.1% in the cognitive domain, 4.0% in the nutritional domain). Among those identified as pre-frail, 0.8% showed impairment in all three domains, 10.5% in two of the three (7.8% in the physical and cognitive domains, 2.2% in the physical and nutritional domains, and 0.5% in the cognitive and nutritional domains), and 68.3% experienced decline in only one domain (39.7% in the physical domain, 25.6% in the cognitive domain, 3.0% in the nutritional domain).

**Table 3:** Results of step one of the PERSSILAA screening showing mean scores and standard deviation

	Group			
	All older adults (*n* = 3764)	Robust (*n* = 2353)	Pre-frail (*n* = 777)	Frail (*n* = 634)
Instrument				
GFI	2.2 (SD ± 2.3)	0.9 (SD ± 1.0)	2.7 (SD ± 1.3)	6.3 (SD ± 1.5)
PF10	80.2 (SD ± 24.0) 19.7% Functional decline	92.0 (SD ± 9.5)	64.7 (SD ± 25.5) 50.4% Functional decline	55.8 (SD ± 29.3) 55.0% Functional decline
AD8	0.7 (SD ± 1.2) 14.1% Cognitive impairment	0.2 (SD ± 0.4)	1.2 (SD ± 1.3) 36.5% Cognitive impairment	1.9 (SD ± 1.9) 46.4% Cognitive impairment
MNA-SF	10.3 (SD ± 1.4) 5.2% Malnutrition	10.8 (SD ± 0.7)	10.2 (SD ± 1.3) 6.5% Malnutrition	9.0 (SD ± 2.2) 22.9% Malnutrition

### Results of step two of the PERSSILAA screening

In total, 623 older adults marked as pre-frail accepted the invitation for step two of the PERSSILAA screening. Table 4 presents the outcomes of the second set of assessments for the physical domain. Based on these outcomes, 69.7% of the pre-frail older adults were considered to have functional decline in the physical domain. The average score on the Q*mci* screen was 65.1 (SD ± 11.3). Based on their Q*mci* screen scores (Table 4), 31.0% of the participants were identified as having probable cognitive decline. Those participants experience especially problems with the task focusing on verbal fluency and logical memory. Finally, two instruments provided extra information on the nutritional domain. The average score on the extended version of the MNA was 26.7 (SD ± 2.1) and none of the participants were classified as having malnutrition. However, only 32% of participants had a normal or healthy weight and 67% were overweight or obese. Table 5 provides an overview of the BMI and waist circumference scores. The average waist circumference was 101.3 (SD ± 14.8). Based on the outcome of step two of the PERSSILAA screening 82.0% of pre-frail participants showed functional decline on at least one of the assessed domains.

**Table 4:** Results of step two of the PERSSILAA baseline screening showing mean scores and standard deviation (SD)

		Pre-frail (*n* = 623)
Physical domain	Instrument	
	TUGT	7.9 s (SD ± 3.1)
	CST	12.3 times (SD ± 4.4)
	CSRT	2.2 cm (SD6.9)
	2MST	123.9 times (SD ± 52.0)
Cognitive domain	Q*mci* screen – total score	65.1 (SD ± 11.3)
	Q*mci* - Orientation	9.7 (SD ± 0.7)
	Q*mci* - Registration	4.5 (SD ± 0.8)
	Q*mci* - Clock drawing	13.6 (SD ± 3.1)
	Q*mci* - Delayed recall	13.7 (SD ± 4.8)
	Q*mci* - Verbal fluency	8.3 (SD ± 3.4)
	Q*mci* - Logical memory	15.4 (SD ± 5.6)
Nutrition	MNA	26.7 (SD ± 2.1)
	Waist circumference	101.3 (SD ± 14.8)

**Table 5:** Results of step two of the PERSSILAA baseline screening showing the BMI classification and the waist circumference

BMI Classification		Percentage	Waist circumference
Underweight	< 18.5	1%	76.9 cm (SD ± 6.8)
Normal (healthy weight)	18.5–25	32%	88.7 cm (SD ± 10.7)
Overweight	25–30	42%	103.4 cm (SD ± 9.6)
Obese	> 30	25%	114.8 cm (SD ± 12.7)

### The merit of the PERSSILAA screening

To investigate the merit of the PERSSILAA screening procedure over the use of a frailty scale in isolation, the classification (frail, pre-frail, robust) of the older adults based on the GFI alone and based on the PERSSILAA screening procedure were compared. Based on the GFI alone, only 8.2% (*n* = 207) of the older adults were classified as pre-frail and 75% (*n* = 2.823) as robust. This compares to the proportion identified after step one of the PERSSILAA screening (frail = 16.8%; pre-frail = 20.6%; robust = 62.3%) which differs significantly (Χ^2^ = 5003.6; *p* < 0.05).

Of the 2823 older adults, classified as robust based on only the GFI alone, 16.6% (*n* = 470) experienced impairment on at least one of the frailty subdomains. Only three older adults experienced impairment in all three domains, 46 experienced impairment in two domains (76.1% in the physical and the cognitive domain; 19.6% in the physical and nutrition domain; and 4.3% in the cognitive and nutrition domain) and 421 experienced impairment in one domain (56.7% in the physical domain; 39.7% in the cognitive domain; and 3.6 in the nutrition domain). Given these results, the PERSSILAA screening is better able to identify those older adults who experience functional decline and are at risk of becoming frail than the GFI alone.

## Discussion

This paper presents the PERSSILAA screening pathway and the outcome of the baseline two-step screening and assessment. Identifying those at risk of developing frailty (i.e. pre-frailty) is important to prevent older adults developing functional decline and becoming frail [7, 8]. In total, among a sample of the older population in the east of the Netherlands, 20.6% older adults appeared to be at risk of becoming frail, based on a large-scale deployment of the PERSSILAA screening procedure. These older adults mainly experienced impairments in the physical and cognitive subdomains and could benefit from effective interventions to reduce the level of frailty [6].

The prevalence of frailty varies enormously in Europe [37] and worldwide [38] and therefore it is difficult to compare our results (16.8% frail, 20.6% pre-frail and 62.3% robust). A systematic review focusing on the prevalence of frailty in community-dwelling older adults presented an overall prevalence of frailty of 10.7 and 41.6% of pre-frailty [38]. More recently, the EU-funded Joint Action on Frailty Prevention (ADVANTAGE) showed that the prevalence of frailty among community-dwellers in European countries varied between 12 and 16% depending on the definition of frailty adopted (physical phenotype versus other definitions, respectively) [39]. Our results are also in line with a more recent paper [40] where the following percentage were reported; 15.1% frail, 33.3% pre-frail and 51.4% robust in a population-based sample of 542 community-dwelling older adults aged ≥65 years living in a metropolitan area in Italy using the FRAIL scale.

The PERSSILAA screening focuses on identifying those older adults who are at risk of becoming frail. This is unique as most screening pathways focus on identifying those older adults who are already frail [9, 14]. The PERSSILAA screening approach is easily applicable in daily clinical practice and identifies physical and cognitive decline as well as malnutrition. From literature we know that pre-frail older adults are more likely to transition back to a robust state than those who are frail [41], though there is insufficient evidence for this at population-level European countries [39]. It has to be proven yet which strategy (screening for pre-frailty or frailty) is more effective in order to reduce the burden of disability, dependence, institutionalization, morbidity and mortality that would be the aim of any frailty screening program.

The elements of pre-frailty of the PERSSILAA screening process are inconclusive and need to be revised. The approach of the PERSSILAA project was to go at least beyond the physical domain and by this first the cognitive and nutritional domain were also assessed during the first and second step of the PERSSILAA screening pathway. As social isolation and loneliness cause severe health problems, a questionnaire on the social domain, as the Loneliness Scale developed by De Jong Gierveld and colleagues [42], should be included in the screening protocol. In literature, both loneliness as social isolation are linked to numerous negative health outcomes [43] comparable to the negative health outcomes of smoking, obesity, lack of exercise and high blood pressure [44].

Given its simplicity, with an emphasis on self-screening using brief paper-based or online questionnaires, the PERSSILAA screening process is potentially useful for primary prevention to define those pre-frail older adults who will benefit most from intervention programmes to build reserve and delay or present frailty [6]. PERSSILAA screening can also support health planning and allocation of limited health and social care resources in the community. It is partially self-administrated and efficient as after the initial triage (step one) it selects out probable pre-frail older adults who are then invited for a face-to-face assessment (step two). The results of the PERSSILAA screening (step 1 and step 2) model were made available to participating municipalities [15] and these results could help them to allocate resources based on objective data. Further, while there is as yet insufficient data to support population-level screening for frailty [14], this study adds to the growing evidence for population-based two-step screening and assessment approaches [14].

Like any study, this one has some limitations. Considering the questionnaires, both the AD8 and MNA-SF are not validated in Dutch and therefore the reliability, validity and sensitivity of these questionnaires for the Dutch population are unknown. Besides, there is a potential for (self) selection bias limiting generalizability. Further, GPs excluded those considered ‘too’ frail, unwell or unsuitable to participate in the screening. Next to this, compared to the general Dutch population the number of high educated older adults seems to be overrepresented and this could have affected the overall response rate. In addition, the response rate was modest with approximately a third of those invited to voluntarily complete the PERSSILAA questionnaire and consenting to participate. This disappointing response rate could be due to the choice to send out the questionnaire via a mass mailing. To increase the response-rate the questionnaires could be to hand out by the GPs. During the participatory design process [15] GPs were not positive about this more practice-based strategy as not all older adults could be reach and handing out the questionnaires was perceived as a burden. The reasons for non-participation are unknown. However, for a subset of participants (*n* = 1228, with a response rate of 30.6%), we asked the non-responders (*n* = 852) to return a postcard when they did not want to participate providing reasons of which 165 (19.4%) postcards were received. These indicated that the main reason for not participating was that they saw no added value as they perceived themselves as being fit (33.3%), or that they thought that participation was not applicable or appropriate from them, as they already received (extensive) medical care (24.2%).

## Conclusions

The PERSSILAA screening pathway outlined in this study is a unique screening process for the primary prevention of pre-frailty that could be utilized by municipalities and GPs to identify community-dwelling older adults who are at risk of becoming frail and to target those who will benefit most from programmes to strengthen their resources and minimize risk factors predisposing to the development of frailty. In addition, the PERSSILAA screening process provides municipalities with objective information to better allocate their resources and potentially support the monitoring and surveillance of frailty at national or transnational level. Further study is required to investigate if this approach could be used with suitable interventions to prevent frailty at population-level [45, 46], and to determine the societal impact of using the approach (the societal return on investment), including GPs’ and patients’ acceptance of the approach.

## Abbreviations

2MST: Two-minute step test
AD8: AD8 Dementia Screening Interview
BMI: Body Mass Index
CSRT: Chair sit and reach test
CST: Chair stand test
GFI: Groningen Frailty Indicator
GP: General Practitioner
MCI: Mild cognitive impairment
MNA: Mini Nutrition Assessment
MNA-SF: Mini Nutrition Assessment Short-Form
n: Number
PERSSILAA: Personalised ICT Supported Service for Independent Living and Active Ageing
PF-10: SF-36 physical functioning scale
Qmci screen: Quick mild cognitive impairment screen
SD: Standard deviation
TUGT: Timed up and go test
